# Antidiarrheal Action of Hydroalcoholic Extract of *Pycnocycla spinosa* in Comparison with Loperamide and Dicyclomine

**Published:** 2011

**Authors:** Hassan Sadraei, Gholamreza Asghari, Mostafa Shams

**Affiliations:** a *Department of Pharmacology, School of Pharmacy and Pharmaceutical Sciences, Isfahan University of Medical Sciences, Isfahan, Iran.*; b *Isfahan Pharmaceutical Sciences Research Center, Isfahan University of Medical Sciences, Isfahan, Iran.*

**Keywords:** Antidiarrheal, Extract, Pycnocycla spinosa, Anti-spasm, Intestine

## Abstract

*Pycnocycla spinosa *Decne. ex Boiss. var. *spinosa *(Fam. Umbelliferae) is an essential oil-containing wild plant growing in central part of Iran. Hydroalcoholic extract of *Pycnocycla spinosa *has antispasmodic and antidiarrheal activity. The aim of this study was to further investigate antidiarrheal and small intestinal transit effect of *P. spinosa *extract for a comparison with loperamide and dicyclomine.

Male mice fasted over night with free access to water, were treated with *P. spinosa *extract, loperamide, dicyclomine or vehicle (p.o.). After thirty min, castor oil was given orally to the animals. In separate groups, magnesium sulphate was given in the beginning and 30 min after the extract or drugs were administered. The onset and number of wet defecation on the absorbent paper was recorded for each animal for 2.5 h. In another group of mice, intestinal transit of charcoal meal after the administration of extract, loperamide or dicyclomine was determined and compared with the control group.

*P. spinosa *extract sharply reduced castor oil and magnesium sulphate induced diarrhea. The extract, in dose of 1 mg/Kg, had antidiarrheal effect similar to loperamide (2 mg/Kg) and with dose of 0.5 mg/Kg, its antidiarrheal action was greater than that of dicyclomine (5 mg/Kg). Unlike dicyclomine, *P. spinosa *extract significantly reduced the small intestinal transit of charcoal meal. However, its inhibitory effect on intestinal transit was less than loperamide.

This study shows that anti-diarrhea l effect of *P. spinosa *extract is similar to loperamide. The inhibition of intestinal propulsion is a most likely mechanism that may account for anti-diarrhea l activity of the extract.

## Introduction

*Pycnocycla spinosa *Decne. ex Boiss., var. *spinosa *(Fam. Umbelliferae) is an essential oil-containing wild plant growing in central part of Iran (1-4). The most abundant constituents identified in the essential oil are geranyl isopantanoate, caryophyllene oxide, *β*-eudesmol, citronellol, elemicin, *ρ*-cymene, citronellyl acetate, *α*-cadinol, nonadecane,sabinene, octanal, *δ*-candinene, methyl eugenol, decanal, trans-*β*-ocimene, limonene, trans-caryophyllene and octadecane, respectively (5). Hydroalcoholic extract of *P. spinosa *is composed of saponin, flavonoid and alkaloid-rich compounds (6). The hydroalcoholic extract is a relaxant of ileum and inhibits the rat ileum contractions induced by acetylcholine, 5-HT (IC_50_ = 13 ± 2.5 mg/mL) and KCl (IC_50_ = 40 ± 7.3 m g/mL) (5). The antispasmodic action of extract is mainly due to the flavonoid and alkaloid-like components in the extract (6). Hydroalcoholic extract of *P. spinosa *inhibited the rat ileum at lower concentration in comparison with its inhibition of rat bladder or uterus contractions (7, 8), which may indicate that *P. spinosa *extract has a relative selective inhibitory action on the ileum. In addition, *P. spinosa *extract was shown to have a dose-dependent antidiarrheal action in doses of 250 µg/Kg, 500 µ g/Kg and 1 mg/Kg in mice (5). Other study showed that intravenous injection of *P. spinosa *extract at doses that inhibits diarrhea has only a transient effect on blood pressure and heart rate (9). General assessment of drug tolerance and behavioral response also indicated that antidiarrheal doses of *P. spinosa *extract have no significant effect on awareness, mood, CNS excitation, muscle tone, reflex, posture, motor coordination and autonomic activity, although, it reduced motor activity at higher doses (10). Therefore, *P. spinosa *extract is relatively potent in inhibiting the ileums contraction and preventing diarrhea in mice and lethal dose testing study shows that it has a good margin of safety (LD_50_ = 140 mg/Kg) (10). Thus, it can be suggested that *P. spinosa *extract can be a suitable remedy for the treatment of diarrhea and gut spasm; however, no comparative study has been done so far. In this research, we had further studies on the antidiarrheal activity of hydroalcoholic extract of *P. spinosa *extract and its effect on small intestinal transit of charcoal meal and compared it with reference drugs loperamide and dicyclomine.

## Experimental

*Extract preparation*

Aerial parts of *Pycnocycla spinosa *were collected in July from Isfahan University campus located on the base of Sofah Mountain in Isfahan (Iran). *P. spinosa *was identified by Mr Mehregan, a botanist at Department of Biology (Isfahan University) and a sample was deposited in the herbarium in the School of Pharmacy (A24). The collected plant was then dried in shade and blended to fine pieces. Hydroalcoholic extract was obtained by percolation using 70% ethanol. The solvent was evaporated and the dried extract was weighted out for calculating the yield. The dried extract had green-brownish colour and the yield was 12.9% (w/w). 

*Animal and solutions*

Male NMRI mice (25-30 g) obtained from Pasture Institute (Tehran) and housed in the School of Pharmacy were used in this study. The animals were randomly allocated to the different treatment groups, dosed with the drug under investigation and treated with diarrhea inducing agent at suitable time.

Dried extract was dissolved in 70% ethanol to make a 10 mg/mL stock solution and then diluted with distilled water to prepare 1 mg/mL solution. Dicyclomine was made up in distilled water (10 mg/mL). Loperamide was made in 70% ethanol (10 mg/mL) and diluted with distilled water afterwards. Appropriate stock solutions were used in such a way that all animals were given 0.5 mL of drug or extract for each appropriate dose. Magnesium sulphate (Merck) was made up as 10% solution in distilled water. All the treatments were given orally by a 1 mL syringe using a curved animal feeding needle for mice (Harvard Apparatus).

*Castor oil induced diarrhea*

All animals were fasted over night with free access to water. The animals were randomly allocated to different treatment groups, dosed with the drug under investigation, and half an hour later, treated with diarrhea inducing agent castor oil. The first group was used as control and received the vehicle (p.o.). The second and the third groups were given 0.5 mg/Kg and 1 mg/Kg of *P. spinosa *extract respectively. The fourth and the fifth groups were treated with the reference drugs, loperamide (2 mg/Kg, p.o.) and dicyclomine (5 mg/Kg, p.o.) respectively. After the administration of castor oil (0.5 mL), the animal were placed on a white absorbent paper under a large glass funnel for 2.5 h and carefully observed for the incidence of diarrhea. 

*Magnesium sulphate induced diarrhea*

A similar protocol, as for castor oil induced diarrhea, was used. Magnesium sulphate was given first (0.5 mL/mouse of 10% solution). Extract or drugs were given half an hour after magnesium sulphate administration.

*Gastrointestinal transit test*

Mice were divided into four groups of animals. The first group was referred to as the control and orally received the vehicle. The second and third group received oral administration of *P. spinosa *extract in the dose of 0.5 mg/Kg and 1 mg/Kg, respectively. The fourth and fifth groups were orally received the reference drugs loperamide (2 mg/Kg) and dicyclomine (5 mg/Kg) respectively. Half an hour after the treatment, individual animals were orally given 1 mL of charcoal meal (3% charcoal in 10% aqueous tragacanth). Thirty min later, the animals were sacrificed and the intestinal distance, moved by charcoal meal from pylorus to cecum, was measured.

*Measurements and statistical analysis*

Diarrhea was scored as the number of watery defecations at appropriate time. The absorbent paper was changed every 15 min and the number of wet defecations was recorded. Gastrointestinal transit was expressed as the percentage of distance that charcoal moved relative to whole length of small intestine. Mean and standard error of mean (SEM) were calculated for each group of results and compared with the control group using unpaired Student’s t-test.

## Results and Discussion

*Castor oil induced diarrhea*

**Figure 1:**
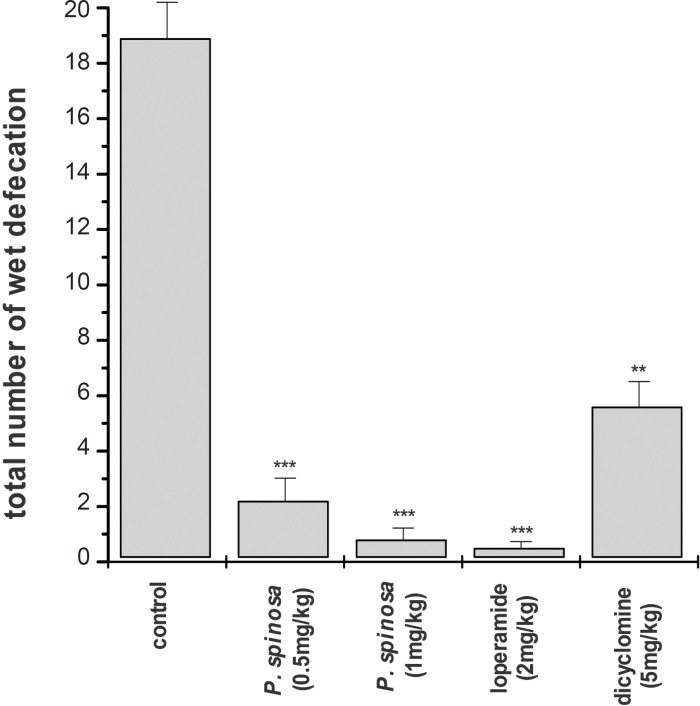
Antidiarrheal activity of *Pycnocycla spinosa *extract on mice with castor-oil-induced diarrhea (0.5 mL, p.o.). Data are mean ± SEM, n = 10 for each group (key **p < 0.01, ***p < 0.001 as compared with control; student’s t-test).

An hour after the administration of castor oil, first sign of diarrhea appeared in control group. The diarrhea severity reached its peak, 10-15 min later and then, gradually subsided. Oral administration of hydroalcoholic extract of *P. spinosa *caused a delay on onset of diarrhea and significantly reduced the number of diarrhea incidence as well as the severity of wet defecation. Over the period of study, 4 out of 10 mice in the group which were treated with 0.5 mg/Kg (p.o) of the extract and 6 out of 10 mice in the group with 1 mg/Kg (p.o) of the extract had no wet defecation at all. These two doses of the extract reduced the total incident of diarrhea by about 85% and 95% respectively in comparison with the control group (Figure 1).

Loperamide was used as a standard antidiarrheal drug and dicyclomine as an antispasmoic drug. Similar to the extract, loperamide delayed the onset of diarrhea and reduced the severity and incidence of diarrhea by about 97% (Figure 1). In the group that was treated with loperamide (2 mg/Kg; p.o), 6 mice out of 10 had no wet defecation over the course of study. The effect of dicyclomine on incidence of diarrhea was less obvious than that of loperamide or *P. spinosa *extract. In the group that was treated with dicyclomine, just one animal was without sign of diarrhea and the number of wet defecations was reduced by 63% as compared with the control group (Figure 1). 

*Magnesium sulphate induced diarrhea*

**Figure 2:**
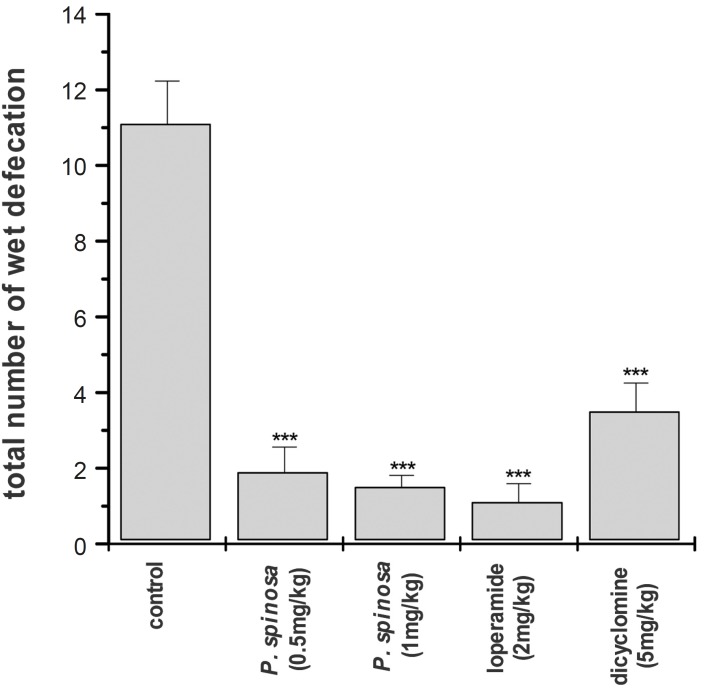
Antidiarrheal activity of *Pycnocycla spinosa *extract on mice with magnesium sulphate (0.5 mL of 10% solution, p.o.) induced diarrhea. Data are mean ± SEM, n = 10 for each group (key ***p < 0.001 as compared with control; student’s t-test).

In the control group, magnesium sulphate caused diarrhea in all animals although the incident of wet defecation over the time was less than those with castor oil. First sign of diarrhea was seen about 45 min after the oral administration of magnesium sulphate and reached to its peak 30-40 min later. Hydroalcoholic extract of *P. spinosa *caused a delay on onset of diarrhea and also reduced the number and the severity of wet defecation (Figure 2). With 0.5 mg/Kg of the extract (p.o) the total incidence of wet defecation was reduced by 83% as compared with the control and 4 animals had no diarrhea at all. In the group that was treated with 1 mg/Kg of the extract (p.o), the incidence of diarrhea was reduced by about 87% with 2 animals without sign of diarrhea. Loperamide (2 mg/Kg) was also effective in inhibiting magnesium sulphate induced diarrhea in mice and its effect was similar to that of extract (Figure 2). In the group treated with loperamide, there was 1 h delay on onset of diarrhea and 6 animals out of 10, showed no sign of diarrhea over the course of study. Dicyclomine (5 mg/Kg) again was less effective than loperamide or the *P. spinosa *extract. In the group which was treated with dicyclomine, the total incidence of diarrhea was reduced by 68% (Figure 2).

*Gastrointestinal transit*

**Figure 3:**
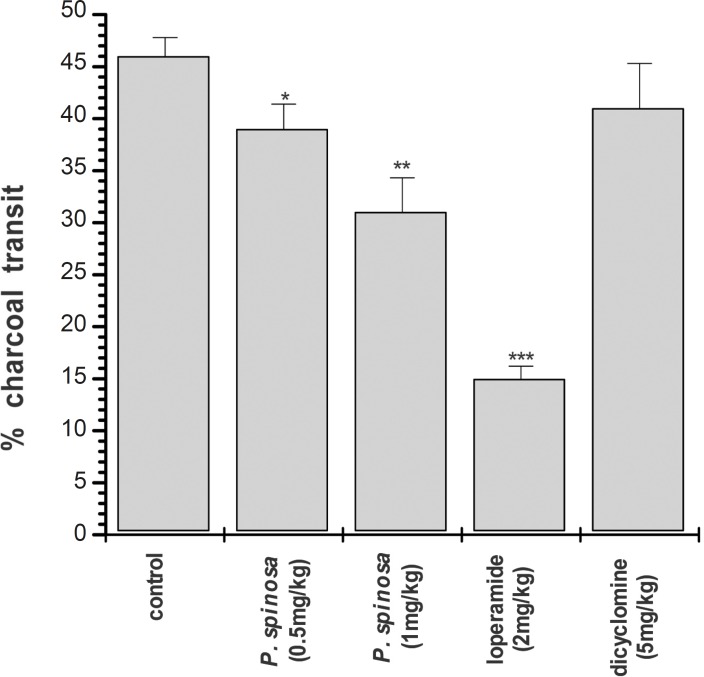
Effect of *Pycnocycla spinosa *extract on small intestinal transit (1 mL of charcoal meal). Data are mean ± SEM, n = 10 for each group (key *p < 0.05, **p < 0.01, ***p < 0.001 as compared with control; student’s t-test).

Hydroalcoholic extract of *P. spinosa *(0.5 mg/Kg and 1 mg/Kg; p.o.) significantly decreased the propulsion of the charcoal meal through the gastrointestinal tract as compared with the control group (Figure 3). The inhibition of intestinal propulsion by loperamide (2 mg/Kg) was greater than the extract whereas the inhibition of intestinal transit by dicyclomine (5 mg/Kg) was not statistically significant (Figure 3).

In this study, castor oil and magnesium sulphate were used as diarrhea-inducing agents. Castor oil cause diarrhea through its active metabolite ricinoleic acid, which stimulates the peristaltic activity of small intestine leading to changes in electrolyte permeability of intestinal mucosa. (11). Magnesium sulphate is an osmotic laxative and induces diarrhea by increasing the volume of intestinal water content due to prevention of water reabsorption. It has reported that magnesium sulphate may promote the liberation of cholecystokinin from the duodenal mucosa. Cholecystokinin increases the secretion and motility of small intestine (11).

The present data shows that in mice, hydroalcoholic extract of *P. spinosa *with doses of 0.5 mg/Kg and 1 mg/Kg prevent castor oil and magnesium sulphate induced diarrhea. The antidiarrheal effect is similar to that of loperamide (2 mg/Kg). The hydroalcoholic extract of *P. spinosa *probably act through decreasing intestinal motility as it was observed that it significantly decreased the intestinal transit of charcoal meal. Antispasmodic action of the hydroalcoholic extract of *P. spinosa *on rat isolated ileum also supports this hypothesis (5, 6). Reduction in gastrointestinal motility assigns more time to the water absorption, although other mechanisms, including the decrease of gastrointestinal secretions cannot be precluded.

Loperamide also significantly reduced diarrhea induced by castor oil and magnesium sulphate, while the inhibition of intestinal transit through charcoal meal with loperamide was greater than the extract. Loperamide is an opioid that acts on presynaptic *μ-*receptor located on cholinergic nerve terminal of gut and thereby, inhibits the gut motility as well as reducing electrolyte and water secretion (11- 13). Opioid delays gastric emptying through acting on gastrointestinal sphincters (11) and that can explain the more pronounced action of loperamide on intestinal transit of charcoal meal. Nevertheless, it is also possible for the inhibitory action of loperamide to have a quicker effect than the hydroalcoholic extract of *P. spinosa*. Dicyclomine is an antagonist of muscarinic receptors with antispasmodic activity on ileum and thereby, inhibits the intestinal contraction (11, 14, 15). The blockade of muscarinic receptors results in a reduction of intestinal motility as well as secretion of water and electrolytes. In this study, dicyclomine inhibited diarrhea induced by castor oil and magnesium sulphate while there was no significant reduction in intestinal transit of charcoal meal. One explanation could be that enough time wasn’t given for full action of the drug to take place or charcoal meal may have reduced the drug action. Unlike atropine, dicyclomine had a relaxant effect on rat isolated ileum contraction induce by KCl at concentrations higher than those which inhibit acetylcholine response (15), suggesting an extra action for dicyclomine on smooth muscle of ileum. Hydroalcoholic extract of *P. spinosa *also inhibits rat isolated ileum contraction induced by KCl (80 mM), acetylcholine and 5-HT (5, 6). It is suggested that the extract action mainly is exerted on ileum smooth muscles. In this regard, there are some similarity between the inhibitory action of dicyclomine and the extract on isolated ileum. As in this study, antidiarrheal activity of the hydroalcoholic extract of *P. spinosa *was more obvious than dicyclomine, it is likely that in addition to inhibiting the ileum peristaltic movement, the extract may also prevent diarrhea via the other mechanism which requires further investigation.

This research confirms the antidiarrheal action of *P. spinosa *extract and when compared with other published data, it is more effective than some other herbal extract remedies that are traditionally being used as antidiarrheal agent (16, 17). Although the constituents of hydroalcoholic extract of *P. spinosa *is not known, the extract contains saponin, flavonoid and alkaloid-like compounds (6). Flavonoid and alkaloid-like constituent account for most of the antispasmodic action of the extract (6) but other unknown ingredients may also play a role.

In conclusion, results of this study are consistent with previous report of antidiarrheal action of hydroalcoholic extract of *P. spinosa *on castor oil induced diarrhea (5). Furthermore, this study also shows that the antidiarrheal action of the hydroalcoholic extract of *P. spinosa *is equipotent with loperamide in two different models of diarrhea. Therefore, further researches for identifying the active ingredient of the extract and other pharmacological factors influencing antidiarrheal activity of the hydroalcoholic extract of *P. spinosa *is recommended.
